# The effect of foot structure and functional foot stability on the gait patterns of the foot

**DOI:** 10.1186/1757-1146-7-S1-A36

**Published:** 2014-04-08

**Authors:** Malia T Ho, John Tan

**Affiliations:** 1 Physical Education and Sports Science, Nanyang Technological University, Singapore 637616, Singapore

## Background

Poor foot structure, such as flat feet or high arched feet were thought to cause excessive foot movement during gait, which in turn is the pre-cursor to foot injuries [1,3].

However, some studies claimed that the differences in gait patterns may not be due to foot structure alone [4,8,11]. It has been suggested that good foot functional stability can ‘protect’ the mal-aligned foot from injuries [9].

Functional Foot Stability is defined in this study as ‘the ability of the foot to continually adjust its position to maintain the body in an upright, balanced position’. An individual with good functional foot stability will be able to sense the foot position and if necessary, correct the position of the foot, thus preventing potential foot injuries.

Whilst studies have been also done to relate foot structure and functional stability [6], as well as functional stability and gait patterns [12,14], no study has been done to investigate the combined effect of foot structure and functional foot stability on gait patterns. Therefore, this study examines the combined effect of foot structure and functional foot stability on running gait patterns.

## Method

Sixty-five subjects (mean age 31 years SD 7.1) had their foot structure scored according to the Foot Posture Index (FPI) [5,10,13] and their functional foot stability was assessed with balance errors scored according to the criteria set out by the Balance Error Scoring System (BESS) [2,7]. Subjects were then put into six groups- Flat foot Stable, Flat foot Unstable, Normal Stable, Normal Unstable, High Arched Stable and High Arch Unstable. The total excursion of the rearfoot, midfoot and first metatarso-phlangeal joints were noted with three dimensional motion analysis. The results were then analysed using ANOVA.

## Results and conclusion

The results showed a significant difference in total excursion of rearfoot inversion/eversion of the flat foot unstable group compared to the other groups.
